# Supplementary material for: Enhancing genetic diversity in Pelargonium: insights from crossbreeding in the gene pool
Source: PeerJ. 2024 Sep 3;12:e17993. doi: 10.7717/peerj.17993 (PMC11378759; doi:10.7717/peerj.17993)
Supplement: Supplemental Information 1 [file peerj-12-17993-s001.docx]

| **Combination** | **Genotype** | **Leaf**  **color** | | | **Leaf**  **size** | | | **Leaf**  **hairiness** | | | | **Flower**  **color** | | | **Flower**  **Stem**  **length** | | | **Flower**  **type** | | | **Flower**  **width** | | | **Flower**  **number** | | | **Bud**  **size** | | | **Earliness** | | | | **Length**  **of**  **internodes** | | | **Stem**  **thickness** | | | **Stem**  **hairiness** | | | | **Stem**  **color** | | | **Growth**  **habit** | | | **Growth**  **vigor** | | | **Plant**  **height** | | |
| --- | --- | --- | --- | --- | --- | --- | --- | --- | --- | --- | --- | --- | --- | --- | --- | --- | --- | --- | --- | --- | --- | --- | --- | --- | --- | --- | --- | --- | --- | --- | --- | --- | --- | --- | --- | --- | --- | --- | --- | --- | --- | --- | --- | --- | --- | --- | --- | --- | --- | --- | --- | --- | --- | --- | --- |
|  |  | **1** | **3** | **5** | **1** | **3** | **5** | **1** | **3** | **5** | **7** | **1** | **3** | **5** | **1** | **3** | **5** | **1** | **3** | **5** | **1** | **3** | **5** | **1** | **3** | **5** | **1** | **3** | **5** | **1** | **3** | **5** | **7** | **1** | **3** | **5** | **1** | **3** | **5** | **1** | **3** | **5** | **7** | **1** | **3** | **5** | **1** | **3** | **5** | **1** | **3** | **5** | **1** | **3** | **5** |
| *P. pel.* 'c1' x *P. pel.* 'c2' | ♀ |  | X |  |  | X |  |  |  |  | X |  |  | X |  | X |  |  | X |  |  | X |  |  | X |  |  | X |  |  |  | X |  |  |  | X |  | X |  |  |  | X |  | X |  |  | X |  |  | X |  |  |  | X |  |
|  | ♂ |  |  | X |  | X |  |  |  | X |  |  | X |  |  | X |  |  | X |  |  | X |  |  | X |  |  | X |  |  |  | X |  |  | X |  |  |  | X |  |  | X |  | X |  |  |  | X |  |  | X |  |  | X |  |
|  | Hybrid | X |  |  |  | X |  |  | X |  |  |  | X |  |  |  | X |  | X |  |  | X |  |  | X |  |  | X |  |  |  | X |  |  |  | X |  |  | X |  | X |  |  | X |  |  |  | X |  |  | X |  |  |  | X |
| *P.* x *hort.* 'c1' x *P. int.* 'c1' | ♀ |  | X |  |  | X |  |  | X |  |  |  | X |  |  | X |  |  | X |  |  | X |  |  |  | X |  | X |  |  |  | X |  |  | X |  |  | X |  |  | X |  |  |  | X |  |  | X |  |  | X |  |  | X |  |
|  | ♂ |  |  | X |  | X |  |  | X |  |  |  | X |  |  | X |  |  | X |  |  | X |  | X |  |  | X |  |  |  |  | X |  |  |  | X | X |  |  |  |  | X |  |  |  | X | X |  |  |  | X |  |  |  | X |
|  | Hybrid |  | X |  | X |  |  |  | X |  |  |  |  | X |  |  | X | X |  |  | X |  |  | X |  |  | X |  |  |  |  | X |  |  |  | X | X |  |  | X |  |  |  |  |  | X |  | X |  |  | X |  |  |  | X |
| *P.* x *hort.* 'c1' x *P.* x *hyb.* 'c1' | ♀ |  | X |  |  | X |  |  | X |  |  |  |  | X |  | X |  |  | X |  |  | X |  |  | X |  |  | X |  |  |  | X |  |  | X |  |  | X |  |  | X |  |  |  | X |  |  | X |  |  | X |  |  | X |  |
|  | ♂ |  |  | X |  | X |  |  |  |  | X |  |  | X |  | X |  |  | X |  | X |  |  |  | X |  |  | X |  |  |  | X |  |  | X |  |  |  | X |  |  |  | X |  |  | X |  | X |  |  | X |  |  | X |  |
|  | Hybrid |  | X |  |  | X |  |  |  |  | X |  |  | X |  | X |  | X |  |  | X |  |  | X |  |  | X |  |  |  | X |  |  |  |  | X |  |  | X |  |  |  | X |  |  | X |  |  | X | X |  |  |  | X |  |
| *P.* x *hort.* 'c1' x *P. zon.* 'c16' | ♀ |  | X |  | X |  |  |  | X |  |  |  | X |  |  |  | X |  | X |  |  | X |  |  | X |  |  |  | X |  |  | X |  |  | X |  |  | X |  |  |  | X |  |  | X |  | X |  |  | X |  |  |  |  | X |
|  | ♂ | x |  | X |  | X |  |  | X |  |  |  |  | X |  | X |  |  | X |  |  | X |  |  | X |  |  |  | X |  |  | X |  |  |  | X |  | X |  |  |  | X |  |  | X |  |  | X |  |  | X |  |  |  | X |
|  | Hybrid |  |  | X | X |  |  |  |  | X |  |  | X |  |  |  | X |  | X |  |  | X |  |  | X |  |  |  | X |  |  | X |  |  |  | X |  | X |  |  |  | X |  |  | X |  | X |  |  | X |  |  |  |  | X |
| *P.* x *hort.* 'c1' x *P. zon.* 'c17' | ♀ | X |  |  | X |  |  |  |  | X |  | X |  |  |  |  | X | X |  |  |  | X |  |  | X |  |  | X |  |  | X |  |  |  |  | X |  | X |  |  | X |  |  | X |  |  | X |  |  |  |  | X |  |  | X |
|  | ♂ |  | X |  | X |  |  |  | X |  |  |  |  | X |  | X |  |  | X |  |  | X |  |  | X |  |  | X |  |  |  | X |  |  |  | X |  | X |  |  | X |  |  | X |  |  | X |  |  |  | X |  |  |  | X |
|  | Hybrid |  | X |  | X |  |  |  | X |  |  |  | X |  |  |  | X |  | X |  | X |  |  |  | X |  | X |  |  |  |  |  | X |  |  | X | X |  |  |  | X |  |  | X |  |  | X |  |  |  |  | X |  |  | X |
| *P.* x *hort.* 'c1' x *P. zon.* 'c20' | ♀ |  |  | X | X |  |  |  | X |  |  |  |  | X |  | X |  |  | X |  |  | X |  |  |  | X |  |  | X |  |  |  | X | X |  |  |  | X |  |  | X |  |  |  | X |  | X |  |  |  |  | X |  |  | X |
|  | ♂ |  | X |  |  | X |  |  | X |  |  | X |  |  |  | X |  |  | X |  | X |  |  |  |  | X |  | X |  |  |  |  | X |  | X |  |  | X |  |  |  | X |  | X |  |  | X |  |  | X |  |  |  |  | X |
|  | Hybrid |  |  | X |  |  | X |  | X |  |  |  |  | X |  |  | X |  | X |  |  |  | X |  | X |  |  |  | X |  |  |  | X |  | X |  |  | X |  |  | X |  |  |  |  | X |  | X |  |  | X |  |  |  | X |
| *P. zon.* 'c1' x *P. zon.* 'c2' | ♀ |  | X |  |  | X |  |  | X |  |  | X |  |  |  | X |  | X |  |  |  | X |  |  | X |  |  | X |  |  |  | X |  |  | X |  |  | X |  |  | X |  |  |  |  | X | X |  |  |  |  | X |  | X |  |
|  | ♂ | X |  |  |  |  | X | X |  |  |  |  | X |  |  | X |  |  |  | X |  |  | X |  |  | X |  |  | X |  |  | X |  |  | X |  |  | X |  |  |  | X |  |  | X |  | X |  |  |  | X |  |  | X |  |
|  | Hybrid |  | X |  | X |  |  |  | X |  |  |  | X |  |  | X |  |  | X |  | X |  |  |  | X |  |  | X |  |  |  |  | X | X |  |  | X |  |  |  |  | X |  |  | X |  | X |  |  |  | X |  |  |  | X |
| *P. zon*. 'c3' x *P. zon.* 'c4' | ♀ | X |  |  |  | X |  |  | X |  |  | X |  |  |  | X |  |  |  | X |  | X |  |  | X |  |  | X |  | X |  |  |  |  | X |  |  | X |  |  |  | X |  | X |  |  | X |  |  |  | X |  |  |  | X |
|  | ♂ | X |  |  |  | X |  |  | X |  |  | X |  |  | X |  |  |  |  | X |  | X |  |  |  | X |  | X |  |  | X |  |  |  | X |  |  | X |  |  |  | X |  | X |  |  |  | X |  |  |  | X | X |  |  |
|  | Hybrid |  | X |  | X |  |  |  | X |  |  | X |  |  |  | X |  |  | X |  |  |  | X |  | X |  |  |  | X |  |  | X |  |  |  | X | X |  |  |  | X |  |  | X |  |  | X |  |  |  | X |  |  |  | X |
| *P. zon.* 'c5' x *P. zon.* 'c6' | ♀ |  | X |  |  | X |  |  | X |  |  | X |  |  |  | X |  | X |  |  |  | X |  |  | X |  |  | X |  |  |  | X |  |  | X |  |  | X |  |  |  | X |  |  |  | X | X |  |  | X |  |  |  | X |  |
|  | ♂ |  |  | X | X |  |  |  |  |  | X |  |  | X |  | X |  |  | X |  |  |  | X | X |  |  |  | X |  |  | X |  |  |  |  | X | X |  |  |  |  |  | X | X |  |  |  | X |  | X |  |  |  |  | X |
|  | Hybrid |  |  | X |  | X |  |  | X |  |  |  |  | X |  |  | X |  | X |  |  |  | X |  | X |  |  |  | X |  | X |  |  |  | X |  |  | X |  |  |  | X |  |  |  | X |  | X |  | X |  |  |  |  | X |
| *P. zon.* 'c7' x L1 | ♀ |  |  | X | X |  |  |  | X |  |  |  |  | X |  | X |  |  | X |  |  | X |  |  | X |  |  | X |  | X |  |  |  |  | X |  | X |  |  |  | X |  |  | X |  |  | X |  |  |  | X |  |  |  | X |
|  | ♂ | X |  |  |  | X |  |  | X |  |  |  | X |  |  | X |  |  | X |  |  | X |  |  | X |  |  | X |  |  | X |  |  |  | X |  | X |  |  |  | X |  |  | X |  |  | X |  |  | X |  |  |  | X |  |
|  | Hybrid |  |  | X |  |  | X | X |  |  |  |  |  | X |  |  | X | X |  |  |  |  | X | X |  |  |  |  | X |  |  | X |  |  |  | X |  | X |  |  |  | X |  |  | X |  |  | X |  | X |  |  |  |  | X |
| *P. zon.* 'c8' x *P. zon.* 'c9' | ♀ | X |  |  |  | X |  |  | X |  |  | X |  |  |  | X |  |  |  | X |  | X |  |  |  | X |  |  | X |  |  | X |  |  |  | X |  | X |  |  |  | X |  |  | X |  |  | X |  |  | X |  |  | X |  |
|  | ♂ | X |  |  |  | X |  |  | X |  |  |  | X |  |  |  | X |  |  | X |  |  | X |  | X |  |  |  | X | X |  |  |  |  |  | X |  | X |  |  |  | X |  |  | X |  | X |  |  | X |  |  |  |  | X |
|  | Hybrid |  |  | X |  | X |  |  | X |  |  | X |  |  |  |  | X |  | X |  |  |  | X |  | X |  |  |  | X |  |  | X |  |  |  | X |  | X |  |  | X |  |  |  |  | X |  | X |  | X |  |  |  |  | X |
| *P. zon.* 'c10' x *P. zon.* 'c11' | ♀ | X |  |  |  | X |  |  | X |  |  |  |  | X |  | X |  |  |  | X |  | X |  |  | X |  |  | X |  |  | X |  |  | X |  |  |  |  | X |  | X |  |  |  | X |  | X |  |  |  | X |  |  |  | X |
|  | ♂ |  | X |  |  | X |  |  | X |  |  |  | X |  |  | X |  |  | X |  |  |  | X |  | X |  |  | X |  |  |  | X |  | X |  |  |  | X |  |  | X |  |  |  | X |  |  | X |  |  | X |  |  | X |  |
|  | Hybrid |  | X |  |  | X |  |  | X |  |  |  | X |  |  |  | X |  | X |  |  | X |  |  | X |  |  | X |  |  |  | X |  | X |  |  |  |  | X | X |  |  |  |  |  | X |  | X |  |  | X |  |  |  | X |
| *P. zon.* 'c12' x *P. zon.* 'c13' | ♀ |  | X |  |  | X |  |  | X |  |  |  |  | X |  | X |  |  | X |  |  | X |  |  |  | X |  | X |  |  |  | X |  |  | X |  |  | X |  |  | X |  |  |  | X |  |  | X |  |  | X |  |  | X |  |
|  | ♂ | X |  |  | X |  |  |  | X |  |  |  | X |  |  | X |  |  | X |  |  |  | X |  | X |  |  | X |  |  |  | X |  |  | X |  |  | X |  |  | X |  |  |  | X |  | X |  |  | X |  |  |  | X |  |
|  | Hybrid |  | X |  |  |  | X |  |  | X |  | X |  |  |  |  | X |  | X |  |  |  | X |  |  | X |  |  | X |  | X |  |  |  | X |  | X |  |  |  | X |  |  |  | X |  | X |  |  | X |  |  |  |  | X |
| *P.* x *hyb.* 'c2' x *P. zon.* 'c14' | ♀ |  | X |  |  | X |  |  | X |  |  |  |  | X |  | X |  |  | X |  |  | X |  |  |  | X |  | X |  |  |  | X |  |  | X |  |  | X |  |  | X |  |  |  | X |  |  | X |  |  | X |  |  | X |  |
|  | ♂ | X |  |  |  | X |  |  | X |  |  |  | X |  |  | X |  |  | X |  |  | X |  |  | X |  |  | X |  |  |  | X |  |  | X |  | X |  |  |  | X |  |  |  | X |  | X |  |  |  |  | X |  | X |  |
|  | Hybrid |  | X |  |  | X |  |  | X |  |  |  | X |  |  | X |  |  | X |  |  | X |  |  | X |  |  |  | X |  |  | X |  | X |  |  | X |  |  |  | X |  |  |  | X |  | X |  |  |  |  | X | X |  |  |
| *P. zon.* 'c15' x *P.* x *hort.* 'c2' | ♀ | X |  |  | X |  |  |  | X |  |  | X |  |  | X |  |  |  |  | X |  | X |  |  |  | X |  | X |  |  | X |  |  |  | X |  | X |  |  |  | X |  |  |  | X |  |  | X |  |  | X |  |  | X |  |
|  | ♂ |  |  | X |  | X |  | X |  |  |  | X |  |  |  | X |  |  | X |  | X |  |  |  | X |  |  | X |  |  |  | X |  | X |  |  |  | X |  |  | X |  |  | X |  |  | X |  |  |  | X |  |  | X |  |
|  | Hybrid |  |  | X | X |  |  | X |  |  |  | X |  |  | X |  |  |  | X |  | X |  |  |  | X |  | X |  |  |  | X |  |  | X |  |  |  | X |  |  | X |  |  | X |  |  | X |  |  |  | X |  |  | X |  |
| *P. zon.* 'c18' x *P. zon.* 'c19' | ♀ |  |  | X |  | X |  |  | X |  |  |  | X |  |  | X |  |  |  | X |  | X |  |  |  | X |  |  | X |  |  | X |  |  | X |  | X |  |  |  |  | X |  | X |  |  | X |  |  | X |  |  |  |  | X |
|  | ♂ |  | X |  | X |  |  | X |  |  |  |  | X |  |  | X |  |  | X |  | X |  |  |  |  | X |  |  | X |  |  | X |  |  |  | X | X |  |  |  | X |  |  | X |  |  | X |  |  |  |  | X |  | X |  |
|  | Hybrid |  |  | X | X |  |  |  | X |  |  |  |  | X |  | X |  | X |  |  | X |  |  | X |  |  | X |  |  |  |  | X |  |  |  | X |  |  | X |  |  | X |  | X |  |  | X |  |  | X |  |  |  |  | X |
| *P. pel.* 'c3' x 'L2' | ♀ |  | X |  |  | X |  |  | X |  |  |  |  | X |  | X |  |  | X |  |  | X |  |  |  | X |  | X |  |  |  | X |  |  | X |  |  | X |  |  | X |  |  |  | X |  |  | X |  |  | X |  |  | X |  |
|  | ♂ |  |  | X |  | X |  |  | X |  |  |  |  | X |  |  | X |  | X |  | X |  |  |  | X |  |  | X |  |  | X |  |  |  | X |  |  | X |  |  | X |  |  |  |  | X |  | X |  | X |  |  |  | X |  |
|  | Hybrid |  |  | X |  | X |  |  | X |  |  | X |  |  |  |  | X |  | X |  |  | X |  |  | X |  |  | X |  |  |  | X |  |  |  | X |  | X |  | X |  |  |  |  |  | X |  | X |  | X |  |  |  |  | X |
| *P. zon.* 'c21' x *P. zon.* 'c22' | ♀ |  | X |  | X |  |  |  | X |  |  | X |  |  |  | X |  |  |  | X |  | X |  |  | X |  |  | X |  |  |  | X |  |  | X |  |  |  | X |  | X |  |  |  | X |  | X |  |  |  | X |  |  | X |  |
|  | ♂ |  | X |  | X |  |  |  | X |  |  | X |  |  |  | X |  |  | X |  |  | X |  |  | X |  |  | X |  |  |  | X |  |  | X |  |  | X |  |  | X |  |  |  | X |  | X |  |  |  |  | X |  | X |  |
|  | Hybrid |  | X |  | X |  |  |  | X |  |  | X |  |  |  |  | X |  | X |  |  | X |  |  | X |  |  | X |  |  |  | X |  |  | X |  |  |  | X |  | X |  |  |  | X |  | X |  |  |  | X |  |  |  | X |
| *P.* x *hort.* 'c3' x 'L3' | ♀ |  |  | X | X |  |  |  | X |  |  |  |  | X |  | X |  |  |  | X |  |  | X |  |  | X |  | X |  | X |  |  |  |  |  | X |  | X |  |  |  | X |  | X |  |  |  | X |  | X |  |  |  | X |  |
|  | ♂ |  | X |  |  | X |  |  | X |  |  | X |  |  |  | X |  |  | X |  |  |  | X |  |  | X |  |  | X | X |  |  |  |  |  | X |  | X |  |  |  | X |  | X |  |  | X |  |  |  | X |  |  | X |  |
|  | Hybrid |  | X |  |  | X |  |  | X |  |  |  | X |  |  |  | X |  | X |  |  |  | X |  |  | X |  | X |  | X |  |  |  |  |  | X |  | X |  |  |  | X |  | X |  |  |  | X |  |  | X |  |  |  | X |
| *P. pel.* 'c4' x *P. gran.* 'c1' | ♀ | X |  |  |  | X |  |  | X |  |  |  | X |  |  | X |  | X |  |  |  | X |  | X |  |  |  | X |  |  |  | X |  | X |  |  |  |  | X |  | X |  |  |  | X |  |  | X |  | X |  |  |  | X |  |
|  | ♂ | X |  |  | X |  |  |  | X |  |  |  | X |  |  | X |  |  |  | X |  | X |  |  |  | X |  | X |  |  |  | X |  |  | X |  |  |  | X |  | X |  |  | X |  |  |  | X |  |  | X |  |  | X |  |
|  | Hybrid |  |  | X | X |  |  |  | X |  |  |  |  | X |  | X |  | X |  |  |  | X |  | X |  |  |  | X |  |  |  | X |  | X |  |  |  |  | X |  | X |  |  | X |  |  | X |  |  |  | X |  |  | X |  |
